# Prevalence of adulteration in dietary supplements and recommendations for safe supplement practices in sport

**DOI:** 10.3389/fspor.2023.1239121

**Published:** 2023-09-29

**Authors:** Andrew R. Jagim, Patrick S. Harty, Jacob L. Erickson, Grant M. Tinsley, Dan Garner, Andrew J. Galpin

**Affiliations:** ^1^ Sports Medicine, Mayo Clinic Health System, La Crosse, WI, United States; ^2^ Exercise & Performance Nutrition Laboratory, Lindenwood University, St. Charles, MO, United States; ^3^ Energy Balance & Body Composition Laboratory, Department of Kinesiology & Sport Management, Texas Tech University, Lubbock, TX, United States; ^4^ BioMolecular Athlete, LLC., Wilmington, DE, United States; ^5^ Department of Kinesiology, Center for Sport Performance, California State University, Fullerton, CA, United States

**Keywords:** dietary supplements, ergogenic aids, inadvertent doping, adulteration, anti-doping rule violation

## Abstract

The prevalence of dietary supplement use among athletes continues to rise with 60–80% of athletes often reporting current or previous use of dietary supplements. While select dietary ingredients have been shown to improve acute performance and enhance training adaptations over time, it is important to still consider the risk vs. reward for athletes before opting to consume a dietary supplement. Previous work has indicated that certain dietary supplements may pose risks for inadvertent doping, may be susceptible to mislabelling, could be banned by certain governing bodies of sport, or pose health risks for certain populations. The purpose of the current narrative review is to summarize the prevalence of adulteration in dietary sport supplement products, outline the risks of inadvertent doping for athletes, and highlight best practices regarding safe supplementation strategies. Analytical studies have found anywhere from 14 to 50% of samples analyzed from dietary supplement products have tested positive for anabolic agents or other prohibited substances. It is important for the consumer to adhere to safe supplementation strategies, which include following serving size recommendations, cross-referencing ingredient profiles with the list of prohibited substances, choosing quality products that have been verified by a third-party certification program, and being cognizant of consuming multiple dietary supplement products with overlapping ingredient profiles. Once these practices have been considered, it is reasonable for an athlete to utilize dietary supplements as a strategy to optimize performance and health, with a low risk of failing a drug test (adverse analytical finding) and experiencing adverse events.

## 1. Introduction

The consumption of dietary supplements (e.g., creatine, beta-alanine, nitrates, etc.) for performance benefits is a popular strategy among athletes. It is estimated that nearly 60%–80% of athletes consume dietary supplements on a regular basis, depending on the specific sport and level of competition (i.e., high school vs. elite) (1–4). Several dietary supplements have been shown to positively influence various aspects of athletic performance, (e.g., strength, muscular endurance, endurance, power, etc.), recovery, and health. Athletes tend to choose dietary supplements under the belief that they will improve performance, enhance recovery, or improve their overall health (5, 6).

In the United States (U.S.), Congress passed the Dietary Supplement Health and Education Act (DSHEA) in 1994, which established an official definition for dietary supplements and designated them as a special category of “foods”. The definition established that dietary supplements are a type of product that is intended to supplement the diet and must contain a “dietary ingredient”, which was subsequently defined as vitamins, minerals, herbs or other botanicals, amino acids, and substances such as enzymes, organ tissues, and glandular extracts. Additionally, these ingredients can include extracts, metabolites, or concentrates of those substances and be found in multiple forms such as tablets, capsules, soft gels, gel caps, liquids, or powders (7). However, recent manufacturing practices in the food industry have begun to blur the lines between dietary supplement products and functional foods, functional beverages, and sports foods (e.g., energy bars, liquid meal replacements, gels, etc.). These products may fall under different regulations and production standards than dietary supplements yet may have similar ingredients as certain dietary supplement products and confer similar benefits (8, 9). Furthermore, each country may have its own definition and regulatory standards of how dietary supplements are defined and regulated. While regulated by the Food and Drug Administration (FDA) in the United States, dietary supplements are not required to submit evidence of safety and efficacy prior to distribution on the open market and rather are subjected to post-market surveillance, particularly when adverse events arise or a failure to comply with Good Manufacturing Practices is identified. However, if a dietary supplement company is proposing the use of a new dietary ingredient, the FDA requires the submission of a “New Dietary Ingredient” notification to inform the FDA of the new ingredients. This submission also includes supporting information that indicates that the new ingredient is reasonably expected to be safe under the conditions of use as recommended or suggested in the labeling (10).

While dietary supplements can offer performance and health benefits, previous work indicates that certain dietary supplements may be susceptible to adulteration, which subsequently may pose risks for inadvertent doping (11, 12). Adulteration is defined as the contamination of a dietary supplement with a prohibited substance (e.g., a doping agent) as established by the doping regulations of the International Olympic Committee and of the World Anti-Doping Agency (11). This can have direct implications on the eligibility of drug-tested athletes as it may predispose them to a doping violation, which would be defined as inadvertent or unintentional doping (12, 13). Further, certain dietary supplements may also be susceptible to mislabelling, may contain ingredients banned by certain governing bodies of sport, or might pose health risks for certain populations (11). Previous reviews have outlined the performance benefits associated with dietary supplements (7, 14) as well as the prevalence of use (2, 15), but few have addressed the risks of adulteration while also providing strategies to mitigate such risks. Therefore, the purpose of the current narrative review is to summarize the prevalence of dietary supplement use among athletes, highlight issues pertaining to adulteration and mislabelling in dietary supplement products, outline the risks of inadvertent doping for athletes, and highlight best practices regarding safe supplementation strategies. Because of the popularity of dietary supplements, it is important to inform athletes, coaches, and sports medicine staff about the risks vs. benefits regarding dietary supplement use and the likelihood of supplements providing ergogenic benefits, while also acknowledging the potential risks, particularly as it pertains to prohibited substances and anti-doping policies.

## 2. Methods

Sources for this narrative review included PubMed, Medline, SPORT Discus, and Google Scholar, with search terms including dietary supplements, ergogenic aids, adulteration, doping, contamination, anti-doping, athletes, and health. Original articles, systematic reviews, meta-analyses, and narrative reviews were included in the articles used for the current review. Additionally, a manual search of the references from the articles identified was also reviewed and subsequently included if they contained pertinent information. Because of the heterogeneity of the articles included in the review, and the multifaceted nature of the information being summarized, a systematic review and meta-analysis were not possible.

## 3. Prevalence of dietary supplement use

The dietary supplement industry has experienced significant growth over the past twenty years; growing at a pace that exceeds the rate at which scientists can determine the safety and efficacy of dietary supplements. In general, athletes report a higher prevalence of use regarding dietary supplements compared to the general population (2). Recent evidence has indicated that approximately 60%–80% of elite athletes report consuming a dietary supplement at some point throughout the year, with various reasons for use ranging from physical performance improvement, improving strength and power, enhancing sports performance, enhancing recovery, and improving health (16). A recent study (15) indicated that approximately 80% of young athletes consumed dietary supplements, with the majority of users (60%) being male. Of those surveyed, sixty percent of athletes reported regularly consuming 2–3 different supplements at the same time and nearly 15% reported using 4 or more different supplements. Parnell et al. (3) reported that out of 567 Canadian athletes (Ages: 11–25 years), 67%, 65%, and 51% self-reported the use of multivitamin/multimineral, vitamin-enriched water, and protein powders, respectively. Performance enhancement and health-related reasons were the most common reasons for supplement use. Supplement use appears to increase with age and with the number of weekly hours spent training, with the exception of masters athletes, which do not report a high prevalence of use (9, 17). It is difficult to determine if level of competition (i.e., high school vs. collegiate, vs. professional) influences dietary intake as there are several confounding factors such as access to education, dietary counseling, budget, sport-type, and country of origin that may all influence the likelihood of an athlete choosing to use dietary supplements (2, 8, 9, 17–21). However, when categorized as elite vs. non-elite, a higher percentage of elite athletes report using dietary supplements compared to their non-elite counterparts (2). Reasons for use appear to change across different age groups with young adults (19–25 year. olds) often reporting the highest percentage of performance enhancement as the primary reason for use compared to younger and older age groups (22). More research is needed to examine how social media and the continued growth of the dietary supplement market have influenced the prevalence of dietary supplement use, particularly with the increased visibility and accessibility of these products for younger athletes. For example, a retrospective study found that while the prevalence of dietary supplement use did not significantly change over an 11-year period from 1998 to 2009, the number of dietary supplements ingested increased from ∼6 to ∼9 per athlete (23).

## 4. Efficacy

A comprehensive summary of dietary supplements that have been shown to be safe and effective within the contextual guise of increasing performance is beyond the scope of this article. In brief, there are several commercially available dietary supplements and functional foods that have repeatedly been shown to be efficacious, well-tolerated, and allowed by most sporting organizations. This list often includes protein (24), carbohydrates (25, 26), creatine (27), caffeine (28), nitrates (29), beta-alanine (30), and sports drinks (14). Readers are directed to previous publications for a more in-depth summary of mechanisms of action along with specific recommendations regarding the safety, efficacy, and dosing guidelines of the aforementioned dietary supplements (7, 14, 31).

## 5. Adulteration and inadvertent doping

### 5.1. Prohibited substances, doping practices, and the world anti-doping code

In an effort to ensure fair competition and prohibit the ingestion or use of prohibited substances in sport, the World Anti-Doping Agency (WADA) serves as the governing authority in most major international competitions as a way to protect the rights of athletes and ensure they are able to participate in doping-free sport (32, 33). It is important for athletes to be aware of substances that do not meet the criteria to be classified as a dietary supplement, which are often ones prohibited by sporting agencies, such as WADA. Moreover, these substances may include pharmaceutical agents, which are commonly classified as Schedule I or Schedule II substances according to the Drug Enforcement Administration within the U.S. (34), thus requiring prescriptions, and not sold over the counter. The International Olympic Committee and WADA maintain an updated list of all prohibited substances and doping methods as part of the World Anti-Doping Code, which includes, but is not limited to, categories comprised of anabolic agents, peptide hormones, growth factors, related substances, mimetics, beta-2-agonists, hormone and metabolic modulators, diuretics and masking agents, stimulants, narcotics, cannabinoids, glucocorticoids, and beta-blockers (32). While some of these prohibited substances have been used in sports for decades, the issue of inadvertent doping and contaminated dietary supplements did not emerge in the world of sports until the late 1990s (33). It is also worth noting that some substances and performance-enhancing strategies or medications are only banned in or around competition time (i.e., within an active competitive season or competition circuit for Olympic qualifying events) and are otherwise allowed to be consumed throughout the year. Certain medical exemptions may also be available for certain qualifying conditions.

### 5.2. Adulteration

Adulteration of dietary supplements can be defined as the accidental contamination or deliberate inclusion (sometimes referred to as “spiking”) of stimulants, anabolic agents, or certain pharmaceuticals, that are included as part of the prohibited substance list mentioned above (35, 36). The first concern to the consumer should be that of their health, particularly if they are unknowingly ingesting these substances. Additional concerns are apparent for those participating in drug-tested sports, as consumption of a prohibited substance, even if unbeknownst to the athlete, could result in a fine, suspension, or ban from the competition if an adverse analytical finding is identified. The World Anti-Doping Agency maintains a strict liability principle in which an anti-doping rule violation occurs whenever a prohibited substance is found in an athlete's bodily specimen (37).

The prevalence of adulterants found in certain dietary supplement products often varies based upon the type of product and target demographic. Dietary supplement products marketed as multi-ingredient pre-workout supplements, *performance-enhancing, anabolic*, or those purported to *promote weight loss* and *fat-burning* often appear to be the ones with the highest prevalence of adulterants found (Table 1). One of the largest analytical studies to date (61) reviewed 634 non-hormonal dietary supplements from 13 different countries and 215 separate suppliers, and found that 94 (14.8%) of the samples contained anabolic androgenic steroids or steroid derivatives at a concentration of 0.01 micrograms per gram to 190 micrograms per gram. Supplements purchased from the Netherlands and Austria contributed to the highest percentage of tainted supplements, representing 25.8% and 22.7% of total tainted supplements from those reviewed. A review from 2008 (62) summarized findings from an international study conducted in 2001/2002, in which authors performed an analysis on 634 nutritional supplements from 13 different countries and found that approximately 15 percent of the non-hormonal nutritional supplements selected (i.e., minerals, vitamins, proteins, creatine, etc.) contained anabolic androgenic steroid-like compounds and prohormones, which were not declared on the label. Later, a 2017 review found rates of contamination between 12% and 58% across 23 studies that met inclusion criteria. Similarly, a recent review (12) found Sibutramine to be the most prevalent (28.34%) prohibited substance found in analyzed samples from dietary supplement products. Sibutramine is a serotonin and norepinephrine reuptake inhibitor originally prescribed as a weight loss medication but was banned in 2019 because of elevated risks for cardiovascular adverse events (12). In total, 248 of the 875 dietary supplements (28.34%) analyzed contained sibutramine, while testosterone and other anabolic steroids were detected in 228 (26.06%), 1,3-dimethylamylamine (DMAA) in 58 (6.62%), fluoxetine in 192 (21.37%), and higenamine in 15 of the 875 dietary supplements (1.71%) analyzed (12). A summary of studies that have provided evidence of adulteration in dietary supplements available on the US market is found in Table 1. Several of the studies reported the detection of various stimulants and anabolic agents in the dietary supplement products analyzed. Pre-workout, thermogenic, and muscle-building products tend to be more susceptible to adulteration. Specifically, several of the products evaluated in the studies included in Table 1 contained various stimulant agents (i.e., ephedrine, methylephedrine, 1–2, -Dimethylamylamine, etc.), steroid and pro-hormone compounds (i.e., testosterone, androstenedione, etc.). As such, athletes consuming these products or products with similar marketing claims should ensure they are selecting products that have been evaluated by third-party organizations to mitigate risks of adulteration and anti-doping violations.

**Table 1:** Evidence of dietary supplement adulteration and mislabeling in the US market.

Author	Supplement inclusion criteria	Included supplement types/claims	Analytical approach	Adulterants identified (prevalence in sample if reported)	Main findings	Reference
Gurley et al. 1998	Nine products which listed Ma-huang (*Ephedra sinica*) as an ingredient.	Weight Loss Thermogenic Energy	HPLC	Ephedrine (100%) Pseudoephedrine (78%) Norephedrine (44%) Norspseudoephedrine (44%) Methylephedrine (44%)	All supplements exhibited significant variability in ephedrine-type alkaloid content	(38)
Green et al. 2001	12 brands of over-the-counter supplements claiming anabolic-androgenic ingredients.	Anabolic-androgenic Benefits	HPLC	Androstenedione (42%) 19-nor-4-androstene-3,17-dione (17%) 4-androstene-3,17-dione (8%) 5-androstene-3,17-dione (8%) 4-androstene-3β, 17β-diol (8%) 5-androstene-3β, 17β -diol (8%) 19-nor-5-androstene-3,17-dione (8%) 19-nor-4-androstene-3β, 17β-diol (8%) 19-nor-5-androstene-3β, 17β-diol (8%) Testosterone (8%)	11 of 12 brands did not meet the labelling requirements of DSHEA.	(39)
Zhang et al. 2012	13 dietary supplements suspected of containing 1,3-Dimethylamylamine.	Energy Thermogenic MIPS	GC	1,3-Dimethylamylamine (DMAA) (100%)	All products contained DMAA which was likely of synthetic origin rather than from natural sources.	(40)
Austin et al. 2014	Seven products which listed 1,3-Dimethylamylamine or a synonym as an ingredient.	Energy Thermogenic MIPS	UHPLC-MS/MS	1,3-Dimethylamylamine (DMAA) (100%)	All products contained DMAA which was likely of synthetic origin rather than from natural sources.	(41)
Cohen et al. 2014a	27 products which met the following criteria: (1) Recalled due to adulteration with pharmaceutical ingredients between January 1, 2009, and December 31, 2012 (2) Available for purchase in July or August 2013 directly from websites of supplement manufacturers or retailers; and (3) The supplement name, manufacturer, and distributor listed on the purchased supplement was identical to the information provided in the FDA recall.	Prohormone Energy Thermogenic Sexual Enhancement	GC-MS or LC-MS/MS	Unspecified anabolic steroid (41%) Sibutramine (19%) Phenolphthalein (11%) Fluoxetine (7%) N-didesmethyl sibutramine (4%) Benzyl sibutramine (4%) Sildenafil (4%) Unspecified aromatase inhibitor (4%)	66.7% of included products were found to still contain banned ingredients in spite of previous FDA recalls.	(42)
Cohen et al. 2014b	Two US-origin samples of a dietary supplement suspected of containing a methamphetamine analog.	MIPS	UHPLC-MS/MS	N,*α*-diethyl-phenylethylamine (ETH) (100%)	The product was found to contain approximately 21–35 mg of the unapproved methamphetamine analog per serving.	(43)
ElSohly et al. 2014	12 products suspected of containing methamphetamine analogs.	MIPS Thermogenic Muscle Recovery	LC-MS/MS	Phenethylamine (25%) N,α-diethyl-phenylethylamine (ETH) (17%)	2 of the 12 products contained the methamphetamine analog ETH, which would likely contribute to potential athlete drug test failures for amphetamines.	(44)
Cohen et al. 2015	14 products which listed ingredients which might refer to a synthetic designer stimulant.	Thermogenic MIPS Energy	UHPLC-MS/MS	1,3-dimethylbutylamine (DMBA) (86%)	The unapproved stimulant DMBA was detected in 12 products, ranging from 13 to 120 mg per serving.	(45)
Attipoe et al. 2016	9 products randomly selected from among the best-selling performance enhancing and weight loss products available on military bases in 2012.	Thermogenic	HPLC-UV or UHPLC-MS/MS	Synephrine Octopamine Cathine Ephedrine Pseudoephedrine Strychnine Methylephedrine	A variety of banned stimulants were found in 8 of the products. Caffeine content varied widely (−7% to +266%) across a nine-month period.	(46)
Cohen et al. 2016	21 products which met the following criteria: (1) Labelled as containing *Acacia rigidula*, which might refer to an amphetamine isomer (2) Available for sale online in the USA between January and April 2014	Nootropic Thermogenic MIPS	LC-QTOF/MS	β-Methylphenylethylamine (BMPEA) (52%)	The unapproved stimulant DMBA was detected in 11 products and was likely of synthetic origin rather than from natural sources.	(47)
Cohen et al. 2017	33 products which were labelled as containing the unapproved stimulant oxilofrine or a synonym.	Energy Thermogenic	LC-QTOF/MS	4-[1-hydroxy-2-(methylamino)propyl]phenol (Oxilofrine) (42%)	Oxilofrine was present in 14 products, with 6 containing pharmaceutical doses or greater.	(48)
Cohen et al. 2018a	24 products labelled as containing the unapproved stimulant higenamine or a synonym.	MIPS Thermogenic Energy	UHPLC-MS/MS and LC-QTOF/MS	Higenamine (100%)	Higenamine was present in all products, with doses ranging from trace amounts to 62 ± 6 mg per serving.	(49)
Cohen et al. 2018b	Six products labelled as containing analogs of the banned stimulant 1,3-Dimethylamylamine.	MIPS Thermogenic	UHPLC-MS/MS	1,4-Dimethylamylamine (50%) 1,3-Dimethylamylamine (33%) 1,3-Dimethylbutylamine (17%) 6-Methyl-2-heptanamine (17%)	All products contained banned or unapproved stimulants.	(50)
Cohen et al. 2018c	12 products which met the following criteria: (1) Labelled as containing *Acacia rigidula*, which might refer to an amphetamine isomer (2) Previously analyzed and reported by Cohen et al. 2016 (3) Available for sale online in the USA in 2017	Nootropic Thermogenic MIPS	LC-QTOF/MS	Oxilofrine (75%) 1,3-Dimethylbutylamine (33%) 1,3-Dimethylamylamine (17%) β-Methylphenylethylamine (8%)	Nine products contained at least one stimulant subject to FDA notices, and 6 contained two or more.	(51)
Zhao et al. 2018	32 products suspected of containing phenylethylamines which listed at least one of the following label claims: “weight loss”, “metabolic rate booster”, “myotrophic agent”, “appetite control/regulation”, “lipogenic”, “lipotrophic”, “thermogenic”, “fat burn”, “burn calories”, “gain strength/intensity”, “stimulant”, “energy aid/booster”, “mental focus”, “positive/uplifting mood”, “ephedra free”, or “acacia”.	Thermogenic Energy	NMR	Phenethylamine (50%) Synephrine (47%) Oxilofrine (38%) Hordenine (19%) β-methylphenethylamine (9%) N-methyltyramine (6%) Octopamine (6%) Deterenol (3%)	Phenylethylamines were detected in 28 of the 32 products.	(52)
Avula et al. 2019	27 products which claimed to contain amines or alkaloid compounds.	Information not available	LC-QTOF/MS	p-Synephrine Isopropylnorsynephrine Picamilon Higenamine Piperine alkaloids β-PEA R-β-methylphenethylamine R-N-benzyl-α-phenethylamine N, N-dimethyl-β-phenethylamine N-methyl 9 diphenethylamine Hordenine 1,3-Dimethylamylamine 1,3-Dimethylbutylamine Omberacetam	67% of the supplements contained undeclared adulterants.	(53)
Cohen et al. 2020a	4 products which claimed to contain the synthetic plant steroid analog 5-alpha-hydroxy-laxogenin.	Information not available	GC-MS and LC-MS/MS	Diosgenin (50%) 5-alpha-hydroxy-laxogenin (25%) Phenibut (25%) Androst-3,5-diene-7,17-dione (25%) β-ecdysterone (25%) 7-keto dehydroepiandrosterone (25%)	A variety of adulterants including an unapproved pharmaceutical and a designer steroid were detected in the products.	(54)
Cohen et al. 2020b	10 products which claimed to contain piracetam, an unapproved nootropic drug.	Nootropic	LC-QTOF/MS	Piracetam (100%)	All products contained piracetam in doses ranging from 831 mg–1,542 mg per serving.	(55)
Cohen et al. 2021a	10 products which claimed to contain either omberacetam, aniracetam, phenylpiracetam, or axiracetam, which are unapproved nootropic drugs.	Nootropic	LC-QTOF/MS	Omberacetam (100%) Aniracetam (20%) Vinpocetine (10%) Phenibut (10%) Picamilon (10%)	The products contained up to 400% of a typical pharmaceutical nootropic drug dose and up to 4 unapproved drugs per product.	(56)
Cohen et al. 2021b	17 products which were labelled as containing deterenol, an unapproved experimental stimulant, or one of its synonyms.	MIPS Thermogenic	LC-QTOF/MS	Deterenol (76%) Phenpromethamine (24%) Oxilofrine (24%) Octodrine (18%) 1,3-Dimethylbutylamine (12%) β-methylphenylethylamine (12%) Higenamine (6%) 1,3-Dimethylamylamine (6%) 1,4-Dimethylamylamine (6%)	9 prohibited stimulants and 8 different mixtures of stimulants were found, with up to 4 experimental stimulants identified in each product.	(57)
Cohen et al. 2022a	9 products which met the following criteria: (1) A previous target of an FDA warning letter due to the presence of β-methylphenethylamine, methylsynephrine, or octodrine. (2) Available for online purchase in January 2022	Thermogenic MIPS	LC-QTOF/MS	Methylsynephrine Octodrine 1,4-Dimethylamylamine Omberacetam	5 of the products were found to contain an FDA-prohibited ingredient, in spite of earlier warning letters.	(58)
Cohen et al. 2022b	7 products which were labelled as containing the unapproved cognitive drug centrophenoxine.	Nootropic	UHPLC-PDA	Centrophenoxine (100%)	Centrophenoxine was present in all products, with doses ranging from 79 to 251 mg per serving.	(59)
Tran et al. 2023	Unspecified number of supplements suspected of containing anabolic steroids and/or prohormones purchased prior to December 2014.	Anabolic-androgenic Benefits	GC-MS, NMR, ESI-LC-MS/MS, LC-MS/MS	17β-hydroxy-2α,17α -dimethyl-5α-androstane-3-one Dimethazine 2,17α-dimethyl-17β-hydroxy-5α-androst-1-en-3-one	Three anabolic steroids were identified in the analyzed products.	(60)

DSHEA, dietary supplement health and education act of 1994; GC, gas chromatography; GC-MS, gas chromatography mass spectrometry; ESI-LC-MS/MS, electrospray liquid tandem mass spectrometry; HPLC, high performance liquid chromatography; HPLC-UV, high performance liquid chromatography with ultraviolet spectroscopy; LC-MS/MS, liquid chromatography-tandem mass spectrometry; LC-QTOF/MS, liquid chromatography-quadrupole time-of-flight/mass spectrometry; MIPS, multi-ingredient pre-workout supplement; NMR, nuclear magnetic resonance spectroscopy; UHPLC-MS/MS, ultra high-performance liquid chromatography tandem mass spectrometry; UHPLC-PDA, ultra high-performance liquid chromatography with photo-diode array.

### 5.3. Inadvertent doping

Inadvertent doping describes the occurrence in which an athlete unknowingly ingests a prohibited substance, most commonly from a dietary supplement, but could also be from food substances, cosmetic products, or other sources of contamination (1, 63). For example, a 2012 study (64) found that athletes returning from competition in China provided urine samples as part of regular anti-doping testing, in which samples contained low amounts of clenbuterol, a sympathomimetic anabolic agent classified as a prohibited substance by WADA. In a follow-up study, 28 volunteers provided urine samples after returning from a trip (average duration of 4–23 days) to China. Clenbuterol was detected in 22 (79%) of the analyzed samples, which suggested possible food contamination. Animal-based food products are more commonly to blame compared to plant-based foods due, in part, to livestock practices in which growth-promoting agents are often provided to animals to maximize the growth, development, or milk production of certain animals (64, 65). It is nearly impossible to determine the prevalence of adulterants found in food products using retrospective analysis. However, recent evidence has reported traces of prohibited substances (beyond a threshold that would elicit an adverse analytical finding) following the consumption of tainted milk products (65). As such, athletes may want to exercise caution when selecting food products both domestically and internationally during competition periods to minimize risks of inadvertent doping. In contrast to inadvertent doping, the practice of doping is defined as one or more of the anti-doping rule violations which include, but is not limited to: (1) The presence of a prohibited substance or its metabolites or markers in an athlete's sample; (2) The use or attempted use by an athlete of a prohibited substance or prohibited method; (3) Possession of a prohibited substance or a prohibited method; Trafficking or attempted trafficking in any prohibited substance or prohibited method: (4) Administration or attempted administration to any athlete in-competition of any prohibited substance or prohibited method, or administration or attempted administration to any outlet out-of-competition of any prohibited substance or any prohibited method that is prohibited out-of-competition; (5) Complicity (e.g., assisting, encouraging, aiding, abetting, conspiring, covering up or any other type of intentional complicity involving an anti-doping rule violation); and (6) Prohibited association as indicated by the World Anti-Doping Code (33, 37, 66). It is worth noting that an athlete may claim they inadvertently ingested a banned substance, despite knowingly using or consuming the prohibited substance, which can be difficult to prove or disprove. In a recent study, it was reported that between 2003 and 2020, in 26% (*n *= 49) of the analytical anti-doping rule violations identified by Anti-Doping Norway, which totaled *n *= 192 during that period, the athlete claimed they had inadvertently ingested a prohibited substance through a dietary supplement ingredient, which had subsequently resulted in the adverse analytical finding (33). Upon further analysis, in 27 of the 49 cases, evidence supporting a causal relationship between the use of a specific dietary supplement and the prohibited substance was detected in the athlete's urine sample. Athletes are often able to appeal an adverse analytical finding and anti-doping rule violation by submitting dietary supplement products they were consuming to be tested for prohibited substances. Because of the widespread use of dietary supplements in sports, and the lack of a full-scale drug testing surveillance system, it is impossible to determine the prevalence of intentional and inadvertent doping, and rather it is limited to select cases of high-profile athletes competing in drug-tested competitions. Adulteration of dietary supplement products likely serves as one of the more common underlying causes of inadvertent doping. Therefore, it is important for athletes to carefully review each product they are consuming.

## 6. Labelling concerns

### 6.1. Mislabelling

Another area of concern regarding the risks of dietary supplements is mislabelling, in which supplement facts labels may not reflect the actual contents of the product, which could also be a reason for the presence of adulterants. An early study by Green et al. (39) found several instances of mislabeling across 12 different dietary supplement products. These included a variety of labelling errors such as several ingredients being listed on the label that were not found, misrepresentation of the amount of ingredients actually present in the product, and failure to declare steroids on the label despite their presence in the product (i.e., adulteration). Additionally, out of the 12 products tested, only one of them contained amounts in the range of 90%–110% of packaging claims. In a study by Desbrow et al. (66) the authors examined samples from multi-ingredient pre-workout supplements and found that the amounts of caffeine actually present ranged from 59% to 176% of packaging claims. Further, the authors found that all but one of the sampled products contained a variation of caffeine within, and between batches, that was considered “practically” significant which was defined as ≥40 mg·serving^−1^ variation. Within the U.S., dietary supplement companies are required to include 100% of the volume of dietary ingredients that are specifically added to the product based on the amount listed on the label; any amount less than this amount would be considered misbranded and in in violation of the law, with the exception of slight deviations that can be determined to be attributable to the analytical method use to quantify contents (67). However, for dietary ingredients that are naturally occurring (e.g., Vitamin C from an herbal extract), the amount must be present at >80% because of inherent variability of the substance's contents (67). Mislabelling practices may pose health risks to the consumer as they may be ingesting far less or greater amounts of a particular ingredient than what is stated on a label, thus misleading to the consumer. It is worth noting that similar issues have also been reported in pre-packaged coffee beverages, in which substantial variations in caffeine content have been found (68). However, coffee is classified as a food and therefore held to different regulatory standards. Regarding dietary supplements, the manufacturer may not be at fault in some of these instances, as they may not be wilfully misrepresenting ingredient contents on their labels, rather it may be the raw ingredient suppliers that could be failing to appropriately mix and package the ingredients. Another potential issue regarding poor quality control with manufacturing practices is the risk of cross-contamination, in which manufacturing equipment is used to produce different products and residue from some ingredients may then cross-contaminant another product being manufactured at the same facility (13, 39). Overall, mislabeling and adulteration of dietary supplement ingredients is an indication of poor-quality control and failure to comply with Good Manufacturing Practices (GMP) in alignment with regulations imposed by the Food and Drug Administration in the U.S.

### 6.2. Proprietary blends

Another labeling concern with dietary supplement products, and one with implications for athletes, is the use of proprietary blends on supplement labels (69). While this practice is designed to protect the intellectual property of the dietary supplement manufacturers, it also precludes the ability to identify the specific quantities of each individual ingredient contained in the blend (69). For example, Jagim et al. (69) reported that among a sample of 100 commercially available multi-ingredient pre workout supplement labels evaluated, nearly half (44.3%) of all ingredients were included as part of a proprietary blend with undisclosed amounts of each ingredient. This may have implications for safety but also in regard to the product's potential efficacy, if under-dosed (69). Within the U.S., the FDA requires that supplement companies list the net weight of the proprietary blend on the supplement facts label, while also providing the full list of ingredients, in descending order of predominance by weight (67). Further, if ingredients do not have established *Daily Values*, this must be noted next to the ingredient with a notation of “Daily Value Not Established”. If a dietary ingredient has a known *Reference Daily Intake* or *Daily Reference Values*, the ingredient must be listed separately, with the individual weight specified (67).

## 7. Adverse events

While limited data exist regarding long-term safety data for several of the dietary supplement ingredients available on the market, some published studies have evaluated short-term safety measures. Additionally, epidemiological studies have published prevalence rates and trends in self-reported adverse event reports (70–73), trends in patient-reported symptoms during Emergency Department visits (74), and trends in poison control calls following adverse events associated with dietary supplement use (75). While the majority of adverse events from dietary supplement use are considered minor medical events (i.e., nausea, gastrointestinal distress, headache, etc.), some of them can be considered major medical events and may require hospitalization or even be life-threating. For example, a commonly reported adverse event or side-effect from creatine supplementation is weight gain, which is often a result from increases in fat-free mass. This may or may not be a desired outcome for athletes, depending on their specific sport and any body composition or training-related goals they may have (76). Conversely, pre-workout and thermogenic supplements may pose a higher risk of adverse events (70). We previously reported that out of 1,045 surveyed multi-ingredient pre-workout supplement users, over half (54%) indicated they had experienced an adverse event following ingestion of the supplement, with skin reactions, heart abnormalities (e.g., rapid heart rate), and nausea being the most common (77). Moreover, after review of adverse events reported to the Food and Drug Administration's Center for Food Safety and Applied Nutrition within the United States, it was reported that thermogenic products had 1.26 times the odds and pre-workout products had 1.75 times the odds of the adverse event being death or life-threatening vs. the less severe outcomes compared with the general dietary supplement (noncaffeinated) group (70). Similarly, during a surveillance period of nine years, which included 15,430 adverse event reports from a similar database, nearly ∼40% of them resulted in a serious outcome, with multivitamins or unspecified minerals serving as the most frequently reported product category resulting in 34.5% of all adverse events (78). However, it is difficult to determine primary causality with certain self-reports or retrospective analyses. Moreover, each individual ingredient likely has its own safety profile and risk of adverse events, which may also be dependent upon the consumer's health history, dietary intake, and concomitant supplement or medication use. Therefore, it is difficult to provide specific statements regarding the safety profiles of all the dietary supplement ingredients commercially available. Furthermore, it is beyond the scope of the current article to provide a comprehensive summary of the risks and adverse events associated with each dietary supplement.

As highlighted in the previous section, energy promoting products, thermogenic products, and muscle-building products tend to have the highest rates of adulteration and adverse events compared to other supplement types available on the US market (12, 50, 61, 62, 70, 79). Therefore, athletes should exercise caution when using these products.

## 8. Safe supplement practices

It is important for the athlete to educate themselves regarding best practices before choosing a dietary supplement product. As with many commercially available products, not all are manufactured with the same level of quality control. There will always be some risk associated with dietary supplement use, however, consumers can mitigate risks by selecting products which have been thoroughly evaluated by third-party verification programs (80). Additionally, it is important for athletes to routinely search the World Anti-Doping Agency's or their respective governing authority's prohibited substances lists (32), as these lists are typically updated annually and provide additional information for other substances or methods that may be banned. However, it is important to note that it may not include all prohibited substances and athletes should exercise caution when ingesting a substance if its status is unknown. It is up to the athlete and their support team to cross-reference the list of prohibited substances (32) with their current dietary supplement products and ingredient profiles to ensure that all ingredients are allowed within that sport. Additionally, for drug tested athletes, it may be worthwhile to retain samples of dietary supplement products and any associated information (lot number or batch number) in the event of a failed drug test and pursuit of an appeal, while also being mindful that the principle of strict liability will still apply.

### 8.1. Rationale for use and adherence to supplementation guidelines

While each athlete's situation is unique, it is important for them to consult with a trained professional (e.g., a registered dietitian nutritionists (RDNs) who specializes in sports dietetics or credentialed Board-certified Specialists in Sports Dietetics (CSSDs)) who has intimate knowledge of the safety and efficacy of dietary supplements (specifically the mechanism of action and rationale for use) in addition to the dietary requirements and physiological demands of the sport (81). Once this has been established, an athlete can determine if a particular dietary supplement may be advantageous for their respective sport, specifically if the primary mechanism of action is relevant to the physiological demands of the sport or if a nutritional deficiency has been identified. Further, it is important to consider the return on investment and pros vs. cons of each dietary supplement ingredient, while considering the potential rationale for use. For example, when possible, athletes may want to undergo laboratory tests to determine blood values for specific mineral or vitamin levels (e.g., Iron, Magnesium, Vitamin D, etc.) prior to supplementing with a specific ingredient. In the event that lab values are normal, a supplement may not be needed and could pose additional risks if continuing with supplementation of certain ingredients as blood levels could rise to a toxic range.

Despite the high prevalence of use, factors influencing dietary supplement use and dietary supplement knowledge of users has not been empirically evaluated on a consistent basis across different populations. Unfortunately, previous research has indicated that athletes rarely consult with a physician, registered dietitians, or content expert prior to consuming dietary supplements (82), and often report “self-education” as a source of information regarding dietary supplements (83). Moreover, athletes tend to overestimate their perceived knowledge of dietary supplements, when compared to objective measures of dietary supplement knowledge (83). Furthermore, athletes may not fully understand the primary mechanisms of action, nor do they closely follow the literature regarding evidence-based guidelines for supplementation strategies (15, 84, 85). As an example, a previous study (85) found that athletes were not able to correctly identify the primary active ingredient in a supplement nor its mechanism of action. Additionally, the athletes failed to identify the recommended dose or commonly reported side-effects for supplements they were consuming (85). Similarly, several athletes among a sample of 247 University student-athletes admitted to knowing little about dietary supplements; however, they did express an interest in wanting to learn more about them (86). While likely not uncommon, this illustrates the need for more education, and the promotion of evidence-based, practical guidelines regarding supplementation strategies for athletes to ensure they are consuming dietary supplements that have been shown to be effective for the desired benefit and safe (and allowed for their sport). Previous reviews by Close et al. (87) and Maughan et al. (14) provide decision-making guides for athletes when considering a dietary supplement. In brief, these guides recommend a food first approach, but not food only in which there may be certain situations in which a dietary supplement can provide ergogenic benefits beyond those attained from food only, even when an optimal food-based diet is consumed (e.g., caffeine, or beta-alanine). Additionally, the authors recommend first establishing a physiological rationale for consuming a dietary supplement, and whether or not sound scientific evidence is available to support its use. Further, a close review of prohibited substances that may be included in the product and whether the product has been evaluated by a third-party organization are crucial steps in this process. Lastly, athletes should be aware of potential adverse effects and any allergies or dietary intolerances to any of the included ingredients before choosing to utilize a dietary supplement.

### 8.2. Poly-supplementation

In clinical settings, physicians are often cautious of poly-pharmacy practices, in which patients may be prescribed a variety (often >5) of pharmaceutical medications to help manage multimorbidity (88). From a clinical standpoint, the concern is that the active ingredients from each of the medications could interact with one another and potentially result in adverse effects or diminished efficacy. As such, a careful review of prescription practices and medication lists are always warranted. Similar caution should be exercised in regard to dietary supplements, in which athletes should pay close attention to the supplement facts information and specific ingredients, and amounts, for each dietary supplement product they may be consuming to avoid the risk of “poly-supplementation” and the consumption of potentially high or dangerous amounts of an ingredient (89). For example, a multi-vitamin, a pre-workout supplement, recovery supplement, energy drink, herbal supplement, and sleep aid may have overlapping ingredients and consumption of all these products within a single day may pose risks for the athlete and could result in adverse effects.

### 8.3. Third-party verification

Lastly, if the decision has been made to consume a dietary supplement, it is important for athletes to select a product from a company that subscribes to rigorous third-party testing and verification. This verification assures the consumer that the product has been tested for purity and quality. Specifically, third-party certification programs typically test for prohibited substances, heavy metals, and labelling accuracy, although the precise testing procedures may vary by organization. Nonetheless, this level of assurance has obvious implications for athletes competing in drug-tested sports and organizations. Furthermore, several sporting organizations, particularly within the U.S., strongly recommend that if teams are providing dietary supplements to their athletes, they should select products that have been verified by a third-party. However, it is currently unknown how effective these recommendations are as more work is needed to support these dietary supplement recommendations. For example, preliminary evidence suggests that ∼20–50% of athletes surveyed (depending on the level of competition) may not consistently use third-party tested supplements (19, 20, 90). However, athletes who receive dietary counseling do appear to make better choices regarding the use of dietary supplements (i.e., choosing more effective supplements), compared to those not receiving counseling (21).

A recent consensus statement (80) outlined the essential features of third-party certification programs to help direct consumers on what to look for when choosing dietary supplement products. The authors note an advantage of third-party certification programs is that they help to deliver a level of transparency and assurance regarding quality control of dietary supplements, both of which help athletes determine the risk vs. reward in regard to deciding on whether or not to consume a supplement. One important component of a third-party tester for athletes is the credentials of the analytical laboratory. In particular, it is important that the analytical lab is compliant with ISO 17,065 standards, which ensures that the laboratory has the required equipment, expertise, process, and methodology to appropriately evaluate the products, and that they do not have any conflicts of interest (80). Compliance with this standard will determine the specific designation of the certification program and which specific products they can appropriately evaluate. This has important implication for athletes who compete in drug-tested sports, to ensure that the specific product they consume is free of prohibited substances. The consensus statement (80) outlined essential features of a third-party verification program, notable recommendations including that the certification program should be accredited to ISO 17,065, the certification program should ensure that manufacturers are registered with the FDA as food facilities, the certifying program should ensure that products are formulated only with ingredients that meet the legal definition of dietary ingredient, and the certifying program performance a toxicological review of all ingredients and formulations, to ensure the levels of ingredients do not exceed the levels recommended by medical associations and/or regulatory authorities (if such recommendations exist). There are limitations of third-party certification programs, which are important to note. Firstly, it is not always possible for these certification programs to test for all of the substances on WADA's prohibited substances list. Moreover, this list is also updated annually, which makes it challenging for certification programs and laboratories to keep up with the evolving list of substances to test for. Some of the more well-known third-party certifications evaluating sports nutrition products are Informed Choice, Informed Sport, NSF Certified for Sport®, the Banned Substances Control Group (BSCG) certifications (e.g., Certified Drug Free®), and Labdoor's Sport certification. Additional organizations, such as US Pharmacopeia (USP), evaluate general health supplements that may also be consumed by athletes. It is imperative that athletes select products that are evaluated by third-party organizations that specifically test for prohibited substances, as not all testing organizations may do so. The large number of certification programs holding different standards makes it confusing for athletes to select the right quality assurance programs to ensure samples are tested for the presence of WADA-prohibited substances (91).

## 9. Conclusions

The consumption of dietary supplements continues to be a popular strategy for athletes who hope to enhance various aspects of performance and health. Regarding the potential efficacy of a dietary supplement, it is critical to first examine training, nutrition, sleep, and lifestyle regimens first as deficiencies in any one of these areas may outweigh benefits of supplementation or may preclude supplements from having any discernable benefit and should be rectified first. Moreover, there should be a conversation between an athlete and their support team regarding the risk vs. reward before making the decision to utilize dietary supplements. In regard to the prevalence of adulteration, research indicates that 10 to 30% of dietary supplements may contain prohibited substances, with common ingredients often including sibutramine, higenamine, 1,3-dimethylamylamine, and androgenic steroid compounds. This poses obvious concerns for drug-tested athletes as they may be at risk of inadvertent doping if they happen to ingest any of the adulterated products and are required to undergo drug testing for their sport. It is important for athletes to select products that have been verified by appropriate third-party certification programs to ensure the quality and purity of the dietary supplement product prior to consumption. Health professionals should encourage athletes to select products evaluated by third-party organizations and follow up on compliance when possible. Additionally, it is important for athletes to cross-reference any products they may be taking (dietary supplements or otherwise) to ensure any of the ingredients are not included on any prohibited substances lists according to their respective sporting authority (e.g., WADA, IOC, Professional Sports Association, NCAA, High School Athletics Association, etc.). When safe supplementation practices are followed, there are several dietary supplements that can offer performance and health benefits, with well-established safety profiles and dosing recommendations, and that can provide ergogenic benefits.
